# Supplementary material for: Content-rich biological network constructed by mining PubMed abstracts
Source: BMC Bioinformatics. 2004 Oct 8;5:147. doi: 10.1186/1471-2105-5-147 (PMC528731; doi:10.1186/1471-2105-5-147)
Supplement: Additional File 2 — The original results of the above study (non-essential files are deleted to keep the file size under the limit set by BMC bioinformatics). [file 1471-2105-5-147-S2.bz2 › chilibotAdditionalFile2/dip05/298ID7486683E1289/html/PIK3R1_PDGFRB.html]

 


 **PIK3R1** and **PDGFRB** 
  
Found 21 abstracts in PubMed, retrieved 05.  
 

 What does Google say? 
 PDF only 
| .edu only 

---

**Interactive relationship** (e.g. stimulation, inhibition, etc)

**Inhibitory relationship**- We describe strategies to i target EGFR, its ligand independent variant EGFRvIII, and PDGFR  [ **PDGFRB** ]  on the cell surface, ii inhibit constitutively activate RAS MAPK and PI3K  [ **PIK3R1** ]  Akt signaling pathways, iii target TP53 mutant tumors, and iv block GBM angiogenesis and invasion.  Ref: 12580545 Brain Pathol, 2003
**Neutral relationship**- Most likely this was due to the presence of a suramin insensitive intracellular PDGFR  [ **PDGFRB** ]  pool that allowed activation of PI3K  [ **PIK3R1** ]  located in intracellular compartments.  Ref: 11473364 J Cell Physiol, 2001
